# Forty Years of Rape Myth Acceptance Interventions: A Systematic Review of What Works in Naturalistic Institutional Settings and How this can be Applied to Educational Guidance for Jurors

**DOI:** 10.1177/15248380211050575

**Published:** 2021-10-26

**Authors:** Lara F. Hudspith, Nadia Wager, Dominic Willmott, Bernard Gallagher

**Affiliations:** 1 University of Huddersfield, UK; 2 Manchester Metropolitan University, UK

**Keywords:** Sexual Assault, Jury Decision-Making, Intervention

## Abstract

A systematic review of research assessing rape myth acceptance (RMA) interventions within institutional settings was conducted. The aim of this review was to inform the development of an educational intervention for jurors in rape trials that addresses rape myths, given previous evidence that RMA can affect decision-making and verdicts (Dinos et al., 2015; Gravelin et al., 2019; Leverick, 2020). 12 databases were searched, filtered to return peer-reviewed journals, published from 1980 to 2020, written in English. After removing duplicates from the 5,093 search results returned, 2,676 studies were screened for inclusion. Research studies were included in the review if they assessed the impact of a naturalistic intervention on RMA within an institutional setting. Studies that did not compare an experimental condition to a control condition or did not randomly allocate participants to conditions were excluded. Studies were also excluded if they used a non-validated, or adapted, RMA measure. 20 Research studies were included within the review and were critically appraised according to an author-created critical appraisal tool. It was concluded that RMA interventions can have a short-term impact upon individuals' RMA. Intervention types that were effective in reducing RMA included those that presented RM information; those that contained an empathy component; and bystander programmes. With regards to duration and format, short interventions led to reductions in RMA, and most successful interventions were presented via videos. Implications for policy and practice, and recommendations for future research, are discussed.

## An Attitude Problem

Conviction rates for rape are low in many countries (Daly & Bouhours, 2010; Jehle, 2012). For example, rape convictions fell to an all-time low in England and Wales in 2020 with only 2.6% of rapes recorded by the police resulting in a conviction (Topping & Barr, 2020). While there are several challenges associated with investigating allegations of rape, which undoubtedly contribute towards low conviction rates, there is said to be an ‘attitude problem’ among many of those working within criminal justice systems (CJS) that also has an impact and acts as a barrier to justice (Temkin & Krahe, 2008; Willmott et al., 2021). Scholars have argued that rape myths (RMs) can inform the views of CJS stakeholders regarding rape, which in turn affects their decision-making and the subsequent progression and outcome of cases. As such, belief in RMs is said to contribute to the disparity between the number of reported rapes and the number of cases that result in convictions, commonly referred to as the *justice gap* (Temkin & Krahe, 2008) or the *attrition problem* (Lees, 2002).

## Rape Myths

The concept of Rape Myths (RMs) was first introduced in the 1970s. Schwendinger and Schwendinger (1974) discussed common false beliefs around rape, calling them ‘sexist myths’ or ‘fallacies’ (p. 18), and Brownmiller (1975) discussed ‘male myths of rape’ describing them as 'distorted proverbs' (p. 312). Examples of myths outlined include the notion that rape can be prevented by verbal or physical resistance, and that women ‘ask for it’ via their actions. Various formal definitions of RMs have now been proposed. Burt (1980) defined RMs as 'prejudicial, stereotyped, or false beliefs about rape, rape victims, and rapists' (p. 217). Further definitions have incorporated the varied damaging functions of such myths. For instance, Lonsway and Fitzgerald (1994) defined rape myths as 'attitudes and beliefs that are generally false but are widely and persistently held,’ adding that they ‘serve to deny and justify male and sexual aggression against women’ (p. 134). Bohner (1998) similarly described them as ‘descriptive or prescriptive beliefs about rape… that serve to deny, downplay, or justify sexual violence…’ (p. 14). As well as having such functions, RMs arguably mold subjective expectations of rape scenarios, culminating in narrow definitions of rape that diverge from the legal definition, and thereby influence stakeholders’ decisions (Ryan, 2011; Temkin & Krahe, 2008).

Brownmiller (1975) and Schwendinger and Schwendinger (1974) highlighted the damaging effects of RMs, arguing that they represent norms that govern women’s actions, and they influence the responses victims received when disclosing assaults, such as victim blaming (Amir, 1967). Brownmiller further argued that the perpetuation of RMs across society increased the incidence of rape.

## Impact of Rape Myths within the Criminal Justice System

The first point at which RMs can influence attrition is the reporting stage. Based on internalized belief in rape myths, victims may not acknowledge their experience of unwanted sex as rape (LeMaire et al., 2016; Reed et al., 2020), which can lead to self-blame, in turn rendering victims reluctant to report to the police (Dardis et al., 2018; Halstead et al., 2017; Orchowski et al., 2009; Weiss, 2010; Zinzow & Thompson, 2011). Victims may also be deterred from reporting to the police if they fear not being believed due to the police accepting RMs (Daly & Bouhours, 2010; Jones et al., 2009; Lorenz et al., 2019; Pearson & Barker, 2018; Sable et al., 2006).

RMs also impact upon reported cases, via the experience of ‘secondary victimisation’ (Orth, 2002; Williams, 1984), or what Lees (1993) termed ‘judicial rape’. That is, victims who perceive the police to be victim-blaming, questioning their credibility, or scrutinizing their character may withdraw their support for the prosecution (Hohl & Stanko, 2015; Jordan, 2001; McMillan, 2018).

RMs also affect both police (Dhami et al., 2018; Hine & Murphy, 2019; O’Neal, 2019; Wentz & Keimig, 2019) and prosecutor decision-making (Beichner & Spohn, 2005; Jordan & Mossman, 2019). Police officers' decisions may be influenced by RMs directly, in terms of their own views, and indirectly due to their expectations of prosecutors' belief in RMs. Similarly, prosecutors' decisions may be influenced by their personal RMA and their anticipation of jurors' beliefs (Daly & Bouhours, 2010; Hohl & Stanko, 2015).

Finally, RMs can have an impact upon jury decision-making in cases that are progressed to trial. Several reviews have reported that it has been consistently shown that RMs can affect judgements of guilt, responsibility, and blame and final verdicts (Dinos et al., 2015; Gravelin et al., 2019; Leverick, 2020). Jurors have also been reported to express views in line with RMs when deliberating their verdicts (Leverick, 2020).

With regards to research concerning genuine trials and jurors, Lundrigan et al. (2019) examined 394 stranger-rape cases to determine whether certain factors could distinguish between convicted and acquitted cases. They concluded that convictions could be predicted by factors concordant with the ‘real rape’ myth (Estrich, 1987), suggesting that jurors may have assessed cases based on expectations held in line with this myth.

## How to Address the Issue of RMs Influencing Jury Decision-Making

As research has consistently shown that RMA has the potential to impact upon jury decision-making, there have been several proposals made as to how this issue could be addressed. Proposals have included screening jurors and excluding those who hold belief in RMs from service (Willmott, 2017; Willmott et al., 2021), the use of judge-only trials (Dripps, 2009; Finn et al., 2011) and the routine introduction of expert witnesses (Office for Criminal Justice Reform, 2006). However, the most recent suggestion made in England and Wales is to present a video regarding RMs to jurors pre-trial (Gillen, 2019; HM Government, 2021), a proposal which is also supported by scholars who have conducted research in this area and have concluded that there is a need to provide jurors with such educational material (Dinos et al., 2015; Willmott et al., 2021).

## Aim of Current Review

It is imperative that the development of any educational materials for jurors is empirically informed. As such, this review was conducted to explore existing interventions designed to challenge RMA, as to provide recommendations for the development of such materials to be used with jurors. Thus, the primary aim of this review was to synthesise research findings from studies that had assessed interventions aiming to reduce RMA. A further aim of the review was to critically appraise the included articles to determine their methodological strengths and weaknesses and provide recommendations for future research evaluating RMA interventions.

This review builds upon the existing literature in several important ways. Previous reviews have been conducted concerning wider rape prevention programmes implemented within universities, which provide some evidence as to the effectiveness of interventions that aim to address RMA. Although such reviews provide valuable insights regarding wider rape prevention programmes, they are limited in several respects. First, such reviews were restricted to incorporating research concerning interventions conducted in university settings only, whereas the current review incorporated all relevant research conducted in any institutional/naturalistic setting. Second, while the wider rape prevention programmes assessed in such reviews have contained a component that address RMA, RMA has often not been the focus of the reviews, as is the case in the current review. For example, in exploring rape prevention programmes implemented within universities, Fellmeth et al. (2013) assessed other variables, such that RMA was not measured in each piece of research included in their review. Third, many such reviews have investigated only one specific program type such as male-only (Wright et al., 2018) or bystander programmes (Jouriles et al., 2018; Katz & Moore, 2013), rather than synthesizing research into several types of programs that included an RMA element. Beyond this, the current review is the first to consider how findings regarding RMA interventions might apply to the court setting in terms of an intervention for jurors in rape trials. Finally, the present review’s inclusion criteria mean that only studies with high scientific rigour are included. As such, the recommendations made are based on the best available evidence.

## Method

A systematic review of research assessing RMA interventions was conducted. Initially, the authors intended to review articles concerning RMA interventions implemented within any setting. This included both naturalistic settings and non-naturalistic settings. Examples of interventions implemented within naturalistic settings included RMA interventions implemented within universities as part of wider university rape prevention programmes, which were being utilized independently of the research being conducted. Non-naturalistic settings included laboratory-based mock-trial studies. It became apparent, however, that research exploring interventions within naturalistic settings was distinct from research exploring RMA interventions provided to mock-jurors within a mock-trial paradigm. For example, mock-trial research often requires the collection and analysis of qualitative data, whereas such data was not seen as pertinent to research conducted within naturalistic settings. Additionally, it was clear that the critical appraisal of research conducted in a naturalistic setting would necessitate consideration of separate criteria to that of mock-trial research. Given the divergence in necessary inclusion and exclusion criteria for articles reporting on naturalistic research conducted in institutional settings compared to research conducted in laboratory settings utilizing a mock-trial paradigm, and the need to utilize separate critical appraisal checklists for the two types of research, the decision was made to produce two separate reviews. The first systematic review is presented here, whereas the systematic review of research concerning mock-trials is presented elsewhere.

Since the initial aim of the review was to explore research conducted in any setting (i.e. naturalistic and laboratory-based/mock-juror paradigms), the search terms produced were initially necessarily broad. Indeed, terms were included concerning mock juries and mock trial simulations. However, such studies were subsequently excluded from the current review as they have been synthesized elsewhere in a second review concerning RMA interventions implemented exclusively within such settings. In addition, numerous general terms were included such as ‘lower*’ to return the majority of relevant results. This was felt necessary as several titles and abstracts did not specifically reference an ‘intervention’ or ‘program’ despite one being implemented.

The following search terms were combined into search strings: rape AND myth(s), belief(s), view(s), attitude(s) and misconception(s); ‘rape supportive’; ‘rape accepting’; AND program*; intervention; address*; reduc*; educa*; chang*; debunk*; prevent*; lower*; decreas*; mock trial; mock simulation; mock jury; and mock juror*.

Twelve electronic databases were then searched. They were selected upon the basis of their content. Databases consisted of psychological, educational, criminal justice or general sources. The databases that were searched were British Education Index, Child Development and Adolescent Studies, CINAHL, Criminal Justice Abstracts, Educational Administration Abstracts, ERIC, MEDLINE, PsycArticles, PsycInfo, PubMed, Scopus and Social Care Online. Searches of titles, abstracts and keywords were made and were filtered such that only peer-reviewed journals, written in English, published between November 1980 (the publication date of Burt’s 1980 RMA paper) and August 2020 were returned.

**Figure 1.**
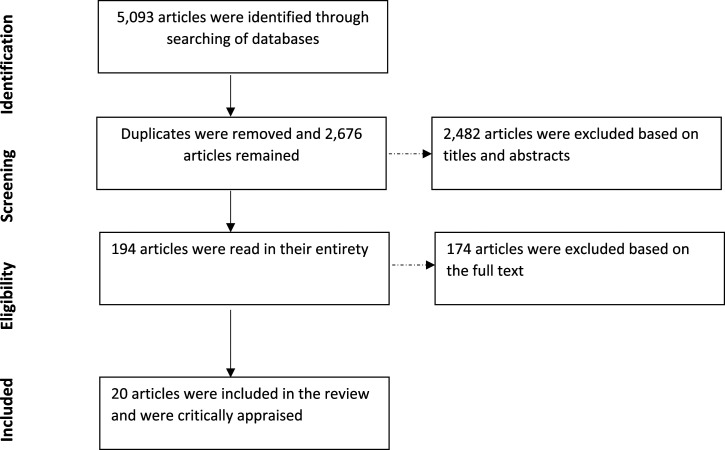
A flowchart to show the screening process undertaken.

Once searches were completed, duplicate results were removed. Articles were then screened based on their titles and abstracts. Articles were included within the review if they concerned research that had assessed an intervention that aimed to reduce RMA that had been implemented within a naturalistic setting. Research that assessed manipulations within an experimental setting, or using a mock-trial design, was therefore excluded from the current review. Research studies were also excluded where no comparison of the intervention and control conditions took place, allocation to conditions was not randomized, non-validated or adapted RMA measures had been used, a specific date rape attitudes measure was used, or where the details of the RMA measure were not clearly reported (Figure 1).

Once the search results were screened as per the inclusion and exclusion criteria, the remaining articles were then read in their entirety. Reference lists of these articles were also checked for potentially relevant articles that were not captured in the searches. Following this, data were extracted from the included articles. Information regarding the content and format of the interventions assessed were collated along with the relevant findings reported within each article (Table 1). The articles were then appraised with the use of an author-created critical appraisal form that comprised relevant criteria from Joanna Briggs checklists Cohort Studies and Randomised Control Trials (Joanna Briggs Institute, 2019). The methodological issues identified during the appraisal process are summarized in Table 2.

**Table 1.** Data extracted from included articles.

Authors	Intervention(s) Content	Intervention(s) Format and Length	Comparison Group Procedure	RMA Measure and RMA Results
Stephens and George (2004)	Theoretical basis not described. Rape statistics, victims’ views, rape trauma information, pornography issues, cultural factors, myth debunking messages, alcohol and the link between sex and violence	Video: Rethinking Rape (Le Page, 1985): a film about acquaintance rape 28 minutes	Video of a mountain climbing documentary	RMAS Intervention had positive impact on non-sexually coercive participants. For sexually coercive participants, there was no difference between the intervention and control video
				
Schewe and O'Donohue (1996)	Theoretical basis not described. Intervention 1 (RSC): Importance of cognitions in rape prevention, sexual communication, rape myths, effects of victimisation and victim blaming. Intervention 2 (VE/OE): Victim empathy and negative consequences for male rapists	Video and behavioural task 50-minute video plus behavioural task (imagining how a victim would feel)	No treatment control	RMAS RSC group RMA significantly lower at post-test than pre-test. No change in RMA for VE/OE or control groups
O’Donohue et al. (2003)	Theoretical basis not described. 3 segments: 1-Debunking rape myths 2-Victim empathy 3-Outcomes discussed to inform accurate outcome expectancies	Video and behavioural tasks (imagining a victim’s perspective and impact parents) 45 minutes	‘The date Rape Flashback’ (Jhally, 1994) as a ‘typical’ rape prevention video: Rape definition, rape as violence not sex and cultural factors	RMAS Experimental intervention more effective than control intervention. High-risk participants responded more favourably to the intervention than low-risk participants
Davis and Liddell (2002)	Theoretical basis not described. Intervention 1 (Traditional): general information regarding acquaintance rape, with video detailing long-term effects. Intervention 2 (socialization-focused): Video clips from TV shows and films demonstrating gendered messages regarding coercive sex. Clips followed by discussion of culture of sexual assault, consent, sexual communication and legal consequences for rapists	Short video clips and discussions 90 minutes	Career development program	RMAS At immediate post-test, intervention 2 group’s RMA was lower than control group’s scores. Scores of intervention 1 group were lowest of all. At 6-week follow-up, RMA of intervention 2 group was not significantly different from that of intervention group 1. No significant differences found between post-test and 6-week follow-up
Senn et al. (2017)	Theoretical basis not described. EAAA (4 stages): 1-Assess: Identifying risk and undermining perpetrator advantages. Counteraction of myths. 2-Acknowledge: Recognizing danger and resisting when in coercive situations. 3-Act: Verbal and physical resistance. 4- Relationships and sexuality: Content from units was incorporated to participants sexual lives	Games, mini-lectures, discussions, group tasks, application and practice using written scenarios, audio clips, video clips and role-play. 12 hours (4 × 3-hour units)	Brochures regarding sexual assault and local resources	IRMA-SF EAAA RMA was significantly reduced at each time point. Effect size diminished over time
Stephens and George (2009)	Theoretical basis: ELM and cognitive dissonance Theory (Festinger, 1957) 1-Introduction showing intervention’s personal relevance to participants 2-Video 3-Introduction to 4 4-Intersection of alcohol and rape on campus	Video: ‘How to Help a Sexual Assault Survivor: What Men Can Do’ (Foubert, 2000) For description, see intervention used by Foubert et al. (2007). 50-minute video and questionnaire	50-minute video regarding the cosmos	RMS and IRMA-SF sleeper effect observed: Significant reduction in RMA for the intervention group compared to control group; however, only at follow-up. Outcomes were mediated by risk of coerciveness level
Banyard et al. (2007)	Theoretical basis not described. Intervention 1 (1 session): Prevalence, causes and consequences of SV. Discussions of bystanders’ role before and after assault. Role-plays of intervening. Creation of bystander plans. Pledges made to be active bystanders. Booster session 2 months later (20-minute group discussion of a 5-minute video depicting a victim asking others for help). Intervention 2 (3 sessions): Expansion of intervention 1. Also followed by a booster session	Unclear – presentation of some form by peer educators Intervention 1: 90 minutes Intervention 2: 3 × 90-minute sessions	No intervention control	IRMA-SF At post-test, RMA is lower for intervention 1 group and intervention 2 group, but not for control, with RMA lower for intervention 2 group than intervention 1 group. Effects maintained at 2- and 4-month follow-ups
Foubert et al. (2007)	Theoretical basis: Belief System Theory (Grube et al., 1994). Overview, rape definitions, The Men’s Program (video; Foubert, 2005), victim empathy, how to help survivors, definition of consent and active bystander training	Video (describes a male police officer’s rape by two violent, heterosexual males) and discussions Approx. 1 hour	Intervention not designed to address variables measured as outcomes	IRMA-SF Those in intervention group who later joined a fraternity had lower RMA at post-test and 7-month follow-up than at pre-test. Difference between experimental and control groups statistically significant at post-test and marginally significant at follow-up
Pinzone-Glover et al. (1998)	Theoretical basis not described. Presentation of rape prevalence statistics and legal rape definition. Completion of ‘Rape Myths and Facts’ worksheet with discussion. Discussion of perpetrators’ behaviours and attitudes, acquaintance rape case examples, and techniques to increase personal safety and prevent rape	Discussion by facilitators and completion of worksheet 50–60 minutes	STD awareness intervention	RMAS There was a main effect of time on RMA.
Hines and Palm Reed (2015)	Theoretical basis not described. Adapted BITB program (included DV and SV and various gendered relationships) 3 sections: 1 – University survey results and empathy-building exercise 2- SV as a continuum 3- Becoming an active bystander: Discussions, role-plays and a pledge to intervene	Presentation 2 hours	Compared the presentation presented by peer educators to the presentation presented by professional educators	IRMA-SF Peer education group’s RMA significantly decreased, whereas that of professional educator’s group did not
Forst et al. (1996)	Theoretical basis not described. Intervention 1: Didactic rape prevention program: Lecture, video (Rape Treatment Centre, 1990) shown of survivors discussing the impact of rape, and Q&A. Intervention 2: Experiential theatre presentation covering risk factors and miscommunication. Participants asked to identify behaviours that could have contributed to the rape portrayed	1-Lecture and video 2-Improvisational theatre and interactive task. 1 hour	No intervention	RMAS Neither intervention led to a reduction in RMA for participants who were victims of forced sex, knew a victim or knew a perpetrator
Yeater et al. (2014)	Theoretical basis not described. 3 sections: 1 – Rape myths and facts 2 – Risk factors and perception 3 – Response strategies	12-chapter self-help book with activities 4–9 hours across 16 weeks	Wait-list control	RMAS No significant findings related to RMA reported
Rau et al. (2010)	Theoretical basis not described. Sexual Assault Intervention Training (SAIT) for men, focused on acquaintance rape of women by men: military definitions of sexual assault and statistics, common rape myths, discussed sexual miscommunication, consent, coercive behaviour, peer pressure and suggestions of how to intervene and support survivors	Lecture and slides 2 × 3-minute discussions, 3 audio dramatizations, 25 minutes of the film ‘When a Kiss Is not Just a Kiss: Sex Without Consent’. Length not stated	Educational video drama. Edited to be as long as SAIT	RMAS and RMS SAIT group had lower post-test RMAS and RMS scores than comparison group. Pre-test to post-test RMA changes significantly greater for SAIT group than comparison group
Rau et al. (2011)	Theoretical basis not described. SAIT for women. Focused on preventing sexual assault by male acquaintances. Provided military definitions of sexual assault. Discussed: Epidemiology and consequences of assault, differences in men and women’s perceptions of sexual situations, and specific risk reduction strategies. Also debunked rape myths and provided suggestions of how to help survivors	Lecture and slides 2 × 3-minute discussions, 3 audio dramatizations, 25 minutes of the film ‘When a Kiss Is not Just a Kiss: Sex Without Consent’. Length not stated	Educational video drama. Edited to be as long as SAIT.	RMAS and RMS significant difference in RMAS and RMS form pre-test to post-test. Effects did not vary by condition
Elias-Lambert and Black (2016)	Theoretical basis not described. BITB: Covered prevalence, causes and consequences of sexual assault. Discussed how participants can prevent sexual assault as active bystanders. Included role-playing how to intervene safely. Participants created bystander plans and pledged to be active bystanders	Includes discussions with facilitators, roleplaying 90 minutes	LGBT program presented by a community LGBT resource centre	IRMA-SF RMA decreased from pre-test to post-test, and from pre-test to follow-up
Foubert and Masin (2012)	Theoretical basis: Belief System Theory (Grube et al., 1994). The Men’s program (Foubert, 2011). For description, see Foubert et al. (2007)	Video and discussions 1 hour	Standard brief given to US soldiers	IRMA Intervention group RMA significantly decreased from pre-test to post-test. RMA of intervention group at post-test significantly lower than comparison group RMA.
Palm Reed et al. (2015)	Theoretical basis not described. Modified BITB: Explicit aim to prevent DV, discussion of both women and men as victims and perpetrators of SV and DV, and SV and DV among LGBTQ+	Presented by co-facilitators. Included an interactive exercise and a discussion. 90 minutes	Traditional Psycho-educational program	IRMA-SF Statistically equal decrease in RMA over time for both groups
Heppner et al. (1995)	Theoretical basis: ELM. Intervention 1: Dating scenarios (Gibson & Humphrey, 1993): Actors portray a rape scene. Participants re-write the script. Actors re-enact the scene incorporating suggestions. Before and after the dramatization actors discuss the issues of intervention 2. Intervention 2: Discussion of rape prevalence, impact of rape, rape myths and gender socialization, and definitions. Video used by Forst et al. (1996). Brief Q&A	Interactional drama presentation compared to discussion and video 90 minutes	Stress management workshop	RMAS Men in intervention 2 had lower RMA than men in the control group at post-test. At group level, there were no differences between any of the groups
Salazar et al. (2014)	Theoretical basis not described. Real consent: 6 interactive modules covering: Informed consent, sexual communication, the roles of alcohol and male socialization in SV, rape victim empathy, and bystander education. Modules also included episodes of a serial drama modelling positive behaviours and positive and negative outcome expectations for intervening and perpetrating SV.	Web-based 6 × 30-minute media-based interactive modules	Health connection: Web-based general health promotion program	IRMA RMA significantly reduced after RealConsent
Johansson-Love & Geer (2003)	Theoretical basis not described. Leaflet with statistics regarding campus rape, how to act in situations in which women are vulnerable and rape myths. Video as used by Forst et al. (1996)	Leaflet and video 6–16 minutes to read a pamphlet and 22-minute video	Leaflet and video regarding sexually transmitted diseases	RMAS RMA of participants in experimental condition significantly lower than that control participants. Effect maintained at 2-week follow-up
Key: RMA – rape myth acceptance; RMAS – Rape Myth Acceptance Scale (Burt, 1980); RMS – Rape Myth Scale (Lonsway & Fitzgerald, 1995); IRMA- Illinois Rape Myth Acceptance Scale (Payne et al., 1999); IRMA-SF – Illinois Rape Myth Acceptance Scale – Short Form (Payne et al., 1999); RSC group: rape-supportive cognitions; VE/EO group: victim empathy/outcome expectancies; EAAA: Enhanced Assess, Acknowledge, Act; BITB: Bringing in the bystander (Banyard et al., 2007; Banyard et al., 2004); ELM – Elaboration Likelihood Model (ELM; Petty & Cacioppo, 1981; 1986a; 1986b); SV – sexual violence; DV – dating violence

**Table 2.** Critical appraisal of included articles.

Authors				Sample				Power Analysis				Baseline Similarity Considered				Potential Confounds Identified & Dealt With				Pre-test				Immediate post-test				Later Follow-up				Attrition Level				Attrition Described and Dealt with			
Stephens and George (2004)				45 UG men Aged 18–25 71% white				No				Not described				Yes, yes				No				Yes				No				N/A				N/A			
Schewe and O'Donohue (1996)				75 (of 225) UG men who scored 15+ on ASA 67% white				No				Yes				Yes, yes				Yes (timing not stated)				No				2 weeks				None				N/A			
O’Donohue et al. (2003)				102 UG men mean age 20 77.5% white				No				Yes				Yes, yes				Yes (timing not stated)				Yes				No				Implied none				N/A			
Davis and Liddell (2002)				90 fraternity men mean age 19.63 95.6% white				No				Not described				Yes, yes				1–2 weeks prior to intervention				Yes				6 weeks				Low				N/A			
Senn et al. (2017)				877 UG women mean age 18.5 73% white				No				Yes				Yes, yes				Immediately prior to intervention				No				1 week, 6, 12 and 18 months				Low				N/A			
Stephens and George (2009)				146 UG men mean age 19.3 all White				No				Not described				Yes, yes				Yes				No				Average of 11.5 days after pre-test and at 5 weeks				High				Described not dealt with			
Banyard et al. (2007)				389 UGs aged 18-23 mean age 19.4 90.4% White				No				Yes				Yes, yes				Yes (timing not stated)				Yes				2, 4 and 12 months				High at follow-ups				Not described, not dealt with			
Foubert et al. (2007)				565 UG men, traditional UG age				No				Not described				Yes, yes				½ participants (timing not stated)				Yes				7 month				Low				N/A			
Pinzone-Glover et al. (1998)				152 UG students aged 18–21 85% White				No				Not described				No, N/A				1week before intervention				No				1 week				Low				N/A			
Hines and Palm Reed (2015)				229 students completed at least one time point mean age 18.11 78.65% White				No				Yes				No, N/A				1 month prior to intervention				No				1 week and 6 months				High				Not described, dealt with			
Forst et al. (1996)				55 UG students aged 19 to 44 mean age 23.9 88% white				Yes				Yes				Yes, yes				Immediately prior to intervention				Yes				2 weeks				Low				N/A			
Yeater et al. (2014)				110 UG women mean age 20 85.5% white				No				Yes				Yes, yes for one and no for another				Yes				No				5 and 16 weeks of 16-week experiment				Medium-high				Not described, not dealt with			
Rau et al. (2010)				1505 US male Navy personnel aged 17–37 65% white				No				Yes				Yes, yes				Yes (½ of participants)				Yes				No				Low				N/A			
Rau et al. (2011)				550 US female Navy personnel aged 17–35 58% white				No				Yes				Yes, yes				Yes (½ of participants)				Yes				No				Low				N/A			
Elias-Lambert and Black (2016)				142 UG fraternity men aged 18–26, mean age 21 47.9% European American				Yes				Yes				No, N/A				Immediately prior to intervention				Yes				5 weeks after intervention				High at follow-up				No significant differences, not dealt with			
Foubert and Masin (2012)				481 US Army males stationed in Germany mean age 25.9 54% white				No				Yes				Yes, no				Immediately prior to intervention				Yes				No				Implied none				N/A			
Palm Reed et al. (2015)				554 UG students Average age 18.1 81.3% white				No				Yes				Yes, yes				Yes (timing not specified)				Time of initial post-test unclear				6 month				High				No significant differences, dealt with			
Heppner et al. (1995)				258 students (50% men, 50% women) 93% white mean age 18.5				No				Yes				Yes, no effect found				5–7 days prior to intervention				Yes				5 months (RMA not measured)				None (high at follow-up)				N/A			
Salazar et al. (2014)				743 male UG students aged 18–24 44.1% white				Yes				Yes				Yes, yes				Immediately prior to intervention				Yes				6 months				High				Described, dealt with			
Johansson-Love & Geer (2003)				151 male students aged 18-39 mean age 20.06 84.5% white				No				Yes				Yes, yes				Immediately prior to intervention (two RMA items only)				Yes				2 weeks				Medium				Not described, not dealt with			
NB: Attrition levels were categorized into low, medium or high, based on the guidance detailed in Schulz and Grimes (2002)																																							

## Results

### Interventions

#### Content

Though there were several distinct components included within each of the interventions, and the interventions were varied in focus and format, several broad clusters of interventions could be discerned based on their content, whilst acknowledging overlap between these groupings for some programs. Common intervention types included bystander training programs, gender-role programs and those that focused on risk-factors and risk-reduction techniques. Most of such programmes also presented general information about rape, typically regarding acquaintance rape of women by men. Such information covered legal definitions of rape and consent, prevalence of rape, and sexual communication issues. Other key components included RM information and victim-empathy training.

With regards to bystander intervention training, such programs aimed to equip participants to feel confident in intervening should they witness sexual violence (SV) or believe SV is about to occur. Participants were taught how to identify potential SV situations and intervene safely. All eight studies that assessed interventions containing bystander training, reported that participants exhibited lower RMA scores post-intervention (Banyard et al., 2007; Elias-Lambert & Black, 2016; Foubert & Masin, 2012; Foubert et al., 2007; Hines & Palm Reed, 2015; Palm Reed et al., 2015; Salazar et al., 2014; Stephens & George, 2009).

Victim-empathy training and information about RMs were also provided within bystander training programs to enhance participants’ motivation to become active bystanders. Such components were also included in other assessed interventions. Overall, assessment of interventions that contained a victim-empathy component produced mixed findings regarding the impact on RMA. O’Donohue et al. (2003) assessed one such intervention, which also provided participants with RM debunking information, reporting that it was successful in reducing RMA. However, Schewe and O’Donohue (1996) found that although an intervention containing an empathy component led to increases in participant’s empathy, it did not impact upon RMA, whereas an intervention that focused on RM information did.

Although victim-empathy training may be an important component of wider rape prevention programmes, such as bystander training initiatives that aim to reduce the occurrence of SV, it may not have a direct impact upon RMA. Rather, in programmes comprising both empathy training and RM information, the RM component may have been instrumental in producing observed RMA reductions. This notion is supported by the fact that eight of the ten assessed interventions that presented specific RM information were reported to be successful in reducing participants’ RMA. Although this finding suggests that direct RM information may be important in reducing RMA, such information presented within a self-help book did not successfully reduce RMA (Yeater et al., 2014).

Finally, research assessing other approaches has produced mixed findings. This is true of both specific gender-socialization–focused interventions and those that incorporated information regarding the link between gender-role socialization and rape. Some authors reported that such interventions reduced RMA (Davis & Liddell, 2002; Salazar et al., 2014), whereas Heppner et al. (1995) did not. Additionally, studies assessing interventions that focused on equipping participants to detect, acknowledge, and avoid ‘risky’ dating behaviours, and interventions that did not focus on risk-reduction but highlighted risk-factors, reported mixed results. Some authors reported that such interventions led to reductions in RMA (Pinzone-Glover et al., 1998; Rau et al., 2011; Senn et al., 2017), whereas others reported they did not reduce RMA (Forst et al., 1996; Yeater et al., 2014).

#### Format

Interventions were delivered in a variety of formats, from traditional teaching methods, namely via face-to-face presentations (typically supplemented by lecture slides and discussions), an interactive web-based program, and a self-help book, to improvisational theatre shows throughout which the actors engaged with participants. Other less interactive methods included presentation of videos, film-clips, and audio-clips. Often, several formats were used within one intervention. The most common format was video. Of the 13 assessed interventions that utilized videos, 11 were reported to have reduced RMA. Importantly, only one of the successful interventions presented a video alone, and 11 of the remaining 12 video interventions were supplemented with interactive tasks. It may be that the effectiveness of a passive video intervention can be enhanced with the incorporation of more interactive tasks such as group discussions.

An interactive, web-based intervention was also reported to have led to reduced RMA (Salazar et al., 2014). These authors noted several benefits of web-based programs, including that they are cost-effective, can potentially reach larger numbers of participants, and the content can be tailored to different groups. Given such benefits of web-based programs, the demonstrated reduction in RMA following this interactive intervention is promising.

Two other interactive methods – a self-help book (Yeater et al., 2014) and programs containing theatrical dramatizations with live actors – demonstrated less success (Forst et al., 1996; Heppner et al., 1995). As both studies assessing theatrical dramatizations were relatively dated, it may be that the interventions were less influential than more recent interventions as the information was at odds with SV norms at the time. Additionally, Forst et al.’s (1996) findings may have been an artefact of the sample used. Forst et al. noted that scores may not have decreased from pre- to post-test owing to most participants having low pre-test scores.

#### Duration and number of sessions

Intervention duration may also impact upon effectiveness. Most interventions (*n* = 17) were 90 minutes or less. Of such interventions, only two were ineffective (Heppner et al., 1995; Forst et al., 1996), demonstrating that even relatively brief interventions can lead to a reduction in RMA. Further, several successful programmes consisted of only one session. Nevertheless, Banyard et al. (2007) found that post-intervention levels of RMA for participants in a three-session condition were lower than those of participants in a one-session condition, thus multiple session programmes may be more beneficial. Banyard et al. also administered follow-up booster sessions to all participants.

#### Intervention presenters

Researchers investigating university-based rape prevention programmes have considered the impact of types of presenters facilitating such programs. Drawing upon attitude change research (Hines & Palm Reed, 2015), such researchers have examined whether peer presenters are more effective presenters than professionals (Paul & Gray, 2011). Eight articles reported the use of peer educators (Banyard et al., 2007; Elias-Lambert & Black, 2016; Foubert et al., 2007; Foubert & Masin, 2012; Heppner et al., 1995; Hines & Palm Reed, 2015; Stephens & George, 2009; Pinzone-Glover et al., 1998). Six specifically stated that peer educators were used, and two reported that doctoral students facilitated the presentation of interventions to undergraduate students. Of the interventions that were presented by peer educators, all but two (Heppner et al., 1995; Pinzone-Glover et al., 1998) led to reductions in participants’ RMA. Heppner et al.’s (1995) intervention may have been ineffective due to the format and measure used, rather than the presenter type. Nevertheless, of the three interventions presented by professional presenters, all but one (Forst et al., 1996) were also reported to be effective. Importantly, the intervention implemented by Forst et al. (1996) may have been ineffective due to other previously discussed issues, such as those concerning the sample. Overall, though, research exploring this factor has not provided definitive findings.

#### Theoretical underpinnings

Four of the articles referred to the assessed intervention’s theoretical basis with regards to attitude change theories (Foubert et al., 2007; Foubert & Masin, 2012; Heppner et al., 1995; Stephens & George, 2009). Several interventions were embedded within the Elaboration Likelihood Model (ELM, Petty & Cacioppo, 1981, 1986a, 1986b), which proposes two routes to attitude change, each associated with a distinct processing style; the ‘peripheral route’, linked with heuristic processing, and the ‘central route’, associated with systematic, thoughtful processing. The model posits that long-term attitude change is more likely when central processing has occurred. Further, the model assumes that the greater the motivation to attend to a message, the more likely individuals are to systematically process, engage with and evaluate it.

Based on the ELM, several factors were incorporated in Heppner et al.’s (1995) improvisational theatre intervention to facilitate systematic processing. A typical dating scenario was used as this was personally relevant to participants and this feature may have increased their motivation to listen to the message. Participants were also required to brainstorm ideas regarding the scenario so as to actively involve them within the session.

Foubert (2020) states that The Men’s Program (Foubert, 2000, 2005, 2011) is based upon both the ELM and Belief Systems Theory (BST, Grube et al., 1994). However, Foubert and Masin (2012), Foubert et al. (2007), and Stephens and George (2009) referred only to BST (Grube et al., 1994) as the theory underpinning the program’s development. BST suggests that interventions must maintain participants’ self-perceptions to produce attitude change. Thus, to do so, participants were approached as potential helpers rather than potential perpetrators, thereby avoiding defensiveness and encouraging co-operation.

Though not in relation to The Men’s Program itself, Stephens and George (2009) also considered the ELM by adding an introduction to their intervention designed to capture participants’ attention and demonstrate that it was of personal relevance to them. Including this information at the outset also allowed for repetition of key information at other time points; this may be important for attitude change given the link between repetition and retention (Hintzman, 2010). Participants were also asked to recall information presented during the intervention, in the belief that this would increase central route processing.

As well as considering the ELM and BST, Stephens and George (2009) considered Cognitive Dissonance Theory (CDT, Festinger, 1957). According to CDT, an individual experiences cognitive dissonance when they hold two contradictory beliefs or are aware that their behaviour is not in line with their beliefs. It is posited that when individuals experience this, they are likely to change either their beliefs or their behaviour. Given this, Stephens and George presented a cognitive dissonance task at the close of their intervention.

Those examining the Men’s Program reported its success, demonstrating some support for the use of BST (Grube et al., 1994). Additionally, Stephens and George (2009) reported a positive correlation between scores on a measure of central route processing and larger RMA changes, supporting use of the ELM. Heppner et al. (1995) reported that although an increase in central route processing was observed, there was no evidence that this led to more lasting RMA change. Nevertheless, the authors suggested that, as the RMA measure could have lacked the sensitivity to detect subtle RMA changes, the results should not be taken to suggest that the ELM intervention is not useful. Finally, the intervention that considered CDT was successful, suggesting it may also be a useful theory to consider when developing interventions.

### Methodological factors

#### Samples

##### Demographics

All studies were conducted in North America, and none used community samples. All samples were relatively homogenous, thus lacking diversity. Seventeen were student samples, eight of which consisted of psychology students only (Forst et al., 1996; Heppner et al., 1995; Johansson-Love & Geer, 2003; Pinzone-Glover et al., 1998; Stephens & George, 2004, 2009; Schewe & O’Donohue, 1996; Yeater et al., 2014). Eleven samples consisted of men only, and three consisted of women only. It was the case that most samples (*n* = 17) comprised only white participants, or white participants represented the largest ethnic group within a sample. The sample ages often ranged from 18–25 years.

##### Sample size and power analyses

Sample size ranged from 45 to 1505. The mean sample size was 361. Only three studies reported using power analyses to determine sample sizes (Elias-Lambert & Black, 2016; Forst et al., 1996; Salazar et al., 2014).

##### Baseline similarity

Five articles did not state whether similarity of participants across conditions was assessed at baseline. All others reported at least one variable that was compared across groups. Reported differences included those in rape empathy, bystander behaviour (Hines & Palm Reed, 2015), RMA, adversarial sexual beliefs (Forst et al., 1996), income (Yeater et al., 2014), history or risk of coerciveness (Rau et al., 2010; Stephens & George, 2009), hostility towards women and SV perpetration (Salazar et al., 2014). The authors did not report controlling for these factors. Rau et al. (2011) reported their study conditions were unequal in terms of the number of participants from each ethnic group, though analyses were conducted to control for this within both studies.

##### Participant characteristics

Several studies explored the moderation effect of the participants’ characteristics. Differences found between participant types included that: male’s RMA changed whereas female’s RMA did not (Heppner et al., 1995); those categorized as non-coercive showed a reduction in RMA, whereas those categorized as coercive did not (Stephens & George, 2004); those at high risk of SV perpetration responded more favourably to interventions than those with low risk (O’Donohue et al., 2003); and that an intervention had less impact on men that were at high risk for using sexually coercive behaviour than those who were at low risk of using such behaviours (Elias-Lambert & Black, 2016). The observed differential impact suggests that tailoring interventions to certain groups may be beneficial.

##### Potential confounds

A range of participant and design factors were identified as potential confounds*.* Participant factors rarely affected outcome variables or were controlled for in analyses. Similarly, regarding design factors, potential order effects were mitigated through counterbalancing in one study (Foubert et al., 2007) and video length was controlled in another (Rau et al., 2011). Nevertheless, three articles did not discuss any potential confounds or approaches to deal with them (Elias-Lambert & Black, 2016; Hines & Palm Reed, 2015; Pinzone-Glover et al., 1998).

One crucial issue to consider when evaluating interventions is the potential for pre-test effects. A pre-test effect is when participants who were pre-tested obtain ‘better’ scores on a post-intervention test than those who were not. Foubert et al. (2007) reported such an effect, demonstrating the importance of acknowledging this as a possibility.

Given the sensitive nature of the views assessed during RMA intervention research, socially desirable responding should also be considered (Edwards et al., 2011). Both Banyard et al. (2007) and Davis and Liddell (2002) reported a correlation of social desirability and RMA, however only Banyard et al. controlled for this.

#### Follow-up periods

##### Time of post-tests

Timing of post-test administration is important as responses in tests presented immediately after an intervention and pre-test may be affected by demand characteristics. Ideally, post-tests should not be administered in the same session as a pre-test, as demonstrated by Davis and Liddell (2002); they found that RMA scores were reduced at an immediate post-test, although scores rebounded for each group including the control. This suggests that participants may be more likely to provide socially desirable responses, or a response that they feel is in line with the experimenter’s hypothesis, at an immediate post-test than they would at a later follow-up. Six articles reported administering immediate post-tests only, whereas the majority administered an additional longer-term follow-up, or a longer-term follow-up alone.

In the context of the present review, the long-term effects of interventions on RMA were not crucial since the aim was to explore the applicability of the findings to the development of educational guidance for jurors, which only necessitates a short-term impact. Nevertheless, long-term follow-ups should be considered when evaluating the efficacy of RM interventions when they are part of primary rape prevention programs, where it is hoped that lower RMA will be associated with favourable behavioural changes or in training professionals, such as the police.

##### Attrition

Of 15 articles that reported a level of attrition, seven reported high levels at one or more time-points. Of such studies, five described the attrition, or reported that either attrition had no effect on outcomes or it was dealt with during analyses (Elias-Lambert & Black, 2016; Hines & Palm Reed, 2015; Palm Reed et al., 2015; Salazar et al., 2014; Stephens & George, 2009), whereas two did not (Banyard et al., 2007; Yeater et al., 2014).

#### Measures

RMA questionnaires used included the R-Scale (Costin, 1985), Rape Myth Acceptance Scale (RMAS; Burt, 1980), Rape Myth Scale (RMS; Lonsway & Fitzgerald, 1995) and Illinois Rape Myth Acceptance Scale/Illinois Rape Myth Acceptance Scale-Short Form (IRMA/IRMA-SF; Payne et al., 1999). Payne et al. (1999) questioned the validity of the RMAS due to the wording used and subsequently developed the IRMA/IRMA-SF. The authors specifically questioned whether the same responses made to RMAS items from different participants may reflect different beliefs as colloquialisms were used and some items covered more than one issue. They also criticized the RMAS for focusing on victims, failing to address other relevant issues such as perpetrators, and because two items assess knowledge of SV statistics rather than attitudes.

The IRMA/IRMA-SF has also been criticized on grounds of its wording (McMahon & Farmer, 2011). Gerger et al. (2007) argued that use of older measures often resulted in large numbers of participants reporting low scores, suggesting that this may be the result of responding based on social desirability due to items being ‘obvious’ and overt. Thus, both the updated IRMA (McMahon & Farmer, 2011) and the Acceptance of Modern Myths about Sexual Aggression (AMMSA; Gerger et al., 2007) were developed to improve the wording of traditional measures, capturing modern, subtler myths. Assessment has shown that the AMMSA is a reliable and valid measure that produces higher means and normally distributed scores (Gerger et al., 2007). A further advantage of the AMMSA is that the sample used during its development was more representative than the student sample employed in developing the updated IRMA. Given this, the language may be applicable to a wider variety of individuals (Schlegel & Courtois, 2019).

#### Comparison conditions

The importance of the type of comparison condition used in evaluations has been raised. The use of an alternative intervention to that under assessment is deemed superior to designs in which no-treatment control conditions are utilized as this latter arrangement allows for factors such as time and social desirability. As such, it increases confidence that observed effects are the result of the intervention itself as opposed to participants merely being in an active treatment condition (Palm Reed et al., 2015). As per the inclusion criteria, each study compared an intervention group to a control group. Further, only one study utilized a no-intervention control group only, and the intervention assessed was not found to be successful (Schewe & O’Donohue, 1996). Thus, the results of this review cannot be said to be based on results that had the potential to be inflated by such a study design.

## Conclusion

As most programs have taken a ‘shotgun’ or package approach, containing a multitude of components, it is not possible to determine which factors are responsible for observed changes in RMA, with regards to content, format and duration (Pinzone-Glover et al., 1998; Paul & Gray, 2011; Schewe & O’Donohue, 1996). It is argued that to further develop the existing evidence base via future research, dismantling designs should be utilized to identify the essential components of RMA interventions (O’Donohue et al., 2003). Hines et al. (2019) adopted such an approach when assessing a bystander intervention. They presented half of their participants with a bystander programme containing an empathy task, and the remaining participants with a bystander programme that did not contain this task, as to determine the effect of the inclusion of this programme component. Conducting further similar research would permit the removal of redundant programme components and thus, could lead to the implementation of more cost-effective and time-efficient approaches. Nevertheless, a summary of the key findings, and their implications, can be provided (Table 3 and 4).

**Table 3.** Critical findings.

Aspect of Interventions	Findings
Content	• Successful programs included those of the following types: those that presented RM information; those that contained an empathy component; those presented as bystander programs. • There was some support for programs concerning gender-roles and risk factors, though findings were mixed
Duration	• Short programs, lasting up to a few hours, can lead to short-term reductions in RMA. • There is some, albeit limited, evidence regarding longer programs that consist of multiple sessions, or a booster session
Format	• Most of the successful interventions were presented via videos, however the majority of successful video interventions were supplemented with interactive tasks such as discussions or a behavioural activity
Presenters	• The limited research comparing different presenter types has produced mixed findings
Theoretical foundations	• There is evidence to suggest that future RMA interventions may be more successful if they are embedded within attitude change theory

**Table 4.** Practice, policy and research implications.

Implication Type	Implications/Recommendations
Practice and policy	• A relatively short intervention that specifically challenges prevalent RMs, delivered using video- or web-based formats could be successful in reducing jurors’ reliance on RMs in the short-term, during trial decision-making. • Several sessions may be more effective than one. Information could be presented after jurors are sworn in and pre-deliberation. • The individual(s) presenting the intervention should be carefully considered. Presenters should be individuals who are regarded as credible or are experts with regards to the information they are sharing. • It is likely to be beneficial for the intervention implemented to be embedded within an attitude change theory.
Research	• Identify key intervention components via dismantling designs, such as that used by Hines et al. (2019), rather than the ‘shotgun’ approaches that have been undertaken thus far. • Explore web-based programs further given their noted benefits and evidence of success. • Compare the delivery of programs utilizing different formats, to assess which format is the most effective. • Compare different levels of interactivity involved in program engagement. • Assess the impact of longer programs and those with multiple sessions. • Determine which attitude change theories have a strong evidence base, to then embed interventions within them. • Use large diverse samples and conduct power analyses to determine sample sizes. • Compare the relative effects of interventions on different participant groups. • Assess baseline similarity as participant characteristics may impact upon results. • Consider and control for potential confounds such as pre-tests (administer post-tests only, ensure sufficient time has elapsed between pre- and post-tests, or utilize Solomon four designs) or socially desirable responding (administer delayed post-tests such that demand characteristics do not lead to inflated results). • Consider follow-ups, and report attrition levels, particularly when assessing behavioural measures. • Use the AMMSA, a subtle measure that may reduce socially desirable responding. This will be particularly useful with non-student samples as other measures developed with American university students, often use language specific to such individuals. • Present an alternative intervention to participants in comparison groups rather than no intervention

Most interventions that incorporated explicit RM information were successful. Bystander training programs were also successful, as were those containing a victim-empathy component. However, bystander programs may have been successful in reducing RMA as they directly addressed RMs. This is concordant with the conclusion of Anderson and Whiston’s (2005) meta-analysis that programs that discussed RMs had more of a positive impact on attitudes towards rape than empathy-focused programmes. Davis and Liddell (2002) also highlighted the importance of explicit RM information as they reported that a program which contained such information lowered RMA more than a gender-socialization programme did. This review also found support for programs concerning gender-role socialization and risk-factors, though findings were mixed.

It can be concluded that short programmes, lasting up to a few hours, can lead to reductions in RMA. However, the impact of implementing longer programmes, perhaps with multiple sessions, should be assessed, particularly with regards to wider rape prevention programmes that aim to lead to long-term attitude and behaviour change.

Most of the successful interventions were presented via videos (*n* = 13); however, the majority of these were supplemented with interactive tasks (*n* = 12). An intervention presented via a web-portal was also successful. As only one web-based intervention was assessed, future research should consider assessing such interventions. Interventions using improvisational theatre presentations (*n* = 2) were unsuccessful, as was the self-help book intervention. Future research should compare programs utilizing formats that differ with regards to their level of interactivity.

There has been limited research investigating the relative effect of different presenter types and it is unclear how findings of research comparing peer to professional presenters can be applied beyond research assessing RMA interventions implemented with university students. Such findings cannot be directly applied to interventions to be used with jurors given that a presenter who would be seen as a peer to one juror may not be considered as such by another. Paul and Gray (2011) argue that it may be of higher importance that presenters are seen as credible by the audience. This should be explored throughout further research.

Finally, the research findings suggest that future RMA interventions may be more successful, if they are embedded within attitude change theory. Future research should determine which theories have a strong evidence-base, to then embed interventions within them.

Based on such conclusions, recommendations can be made regarding the design of a program for jurors. First, a relatively short intervention that specifically challenges prevalent RMs, delivered using video or web-based formats, could be successful in reducing their reliance on RMs in the short-term during trial decision-making. Second, as many of the effective interventions that utilized videos were also supplemented with interactive tasks, using a format with elements of interactivity rather than presenting a video for jurors to passively watch, could also be explored. Third, although a short intervention has the potential to be successful, as a program with several sessions was shown to be more effective than one with only one session, presenting information both after jurors are sworn in and pre-deliberation could be considered. Fifth, the individual(s) presenting the intervention should be carefully considered. Presenters should be individuals who are regarded as credible or are experts with regards to the information that they are sharing. Sixth, it is likely to be beneficial for the intervention implemented to be embedded within an attitude change theory. Although it is expected that interventions will reduce the impact of RMs on individual decision-making, they will not entirely eliminate the problem as jurors who strongly endorse RMs may influence those who do not by introducing such rape mythology during deliberation (Munro, 2019).
